# The Story of the Insane from Year to Year

**Published:** 1890-10-25

**Authors:** 


### THE STORY OF THE INSANE FROM YEAR TO YEAR.

[Annual Reports, Pamphlets, &c., should be addressed to THE EDITOR, The Lodge, Porchester Square, London, W.]

### WARWICK COUNTY ASYLUM.

The committee of visitors begin their report by informing the County Council that there is an urgent need of a detached hospital for infectious diseases, and they think that an expenditure of about £2,000 will suffice. This is rather a low estimate, and the visitors will probably find that a larger sum will be spent on the hospital. At the same time, it is nearer the mark than the North Riding estimate of £4,500 for a similar building. We learn from Dr. Miller's able report that he had 659 patients under his care at the end of 1889. During the year 144 patients were admitted, 92 were discharged, and 77 died. We notice that among those admitted were 12 idiots, 9 epileptics, 6 paralytics, and 37 other incurable cases, making a total of 64 hopeless cases. These would form a nice nucleus of "recent cases" for an acute hospital, wherein we are gravely assured the percentage of recoveries will be something fabulous. The asylum does not seem to be in a satisfactory sanitary condition. Thirteen of the deaths were owing to dysentery and 12 to consumption. There were 17 cases of enteric fever, but, fortunately, no one died of it. Many of the nurses were stricken with the disease, and there was a total absence from duty of 144 weeks during the year. There were also 13 cases of whooping cough. Verily Dr. Miller must have had his hands full, and we hope the year 1890 will be kinder to him.

### DEVON COUNTY ASYLUM.

The year 1889 ended with 923 patients' names on the books of this asylum. There were altogether under treatment during the year 1,059 cases. And this may be said to be the extreme limit which no asylum should ever exceed. Medical superintendents, visitors, commissioners, and home secretaries should unite and absolutely prohibit enlargements where the numbers border on 1,000 beds. One hundred and seventy cases were admitted; 74 were discharged, and 62 died. Of the admissions 31·91 per cent. recovered, and the deaths were 6·85 on the average number resident. The percentage of recoveries is not high, but it is evident, from the analysis of the admissions which Dr. Sanders gives at page 12, that a great many were utterly hopeless on admission, and it is sad to think that of those remaining on the 31st December not more than eighteen of the total number in residence were deemed curable.

### LANCASHIRE COUNTY ASYLUM AT PRESTWICH.

The medical superintendent of this, the largest county asylum in England, has not published his report for 1889; but we gather from the reports of the visitors and the Commissioners, and from the statistical tables, that the asylum contained the enormous number of 2,314 patients, and that the total number under treatment during the year exceeded 3,000. The admissions reached 761, the discharges 508, and the deaths 259. The percentage of recoveries was nearly 37 on the admissions, and the deaths 8·40 on the average number resident. The Commissioners think that some separate provision should be made for the idiot children, and they suggest that one o the four Lancashire asylums should provide special wards for the treatment of these children—a suggestion, which, it is to be hoped, will soon be acted on. The committee express their regret that Mr. Ley, the medical superintendent, was violently assaulted by a discharged attendant. Everyone connected with our county asylums will join with the committee in looking forward to the complete recovery of Ley.

